# Parathyroid hormone type 1 receptor regulates osteosarcoma K7M2 Cell growth by interacting with angiotensinogen

**DOI:** 10.1111/jcmm.16314

**Published:** 2021-01-28

**Authors:** Shenglong Li, Fei Liu, Yi Pei, Yujin Dong, Yaohua Shang

**Affiliations:** ^1^ Department of Bone and Soft Tissue Tumor Surgery Liaoning Cancer Hospital & Institute Cancer Hospital of China Medical University Shenyang China; ^2^ Department of Hand and Foot Surgery Dalian Municipal Center Hospital Affiliated of Dalian Medical University Dalian China

**Keywords:** AGT, CCL9, osteosarcoma, PTHR1

## Abstract

This study aimed to determine the interactions between parathyroid hormone type 1 receptor (PTHR1) and angiotensinogen (AGT) and the effects of these agents on osteosarcoma (OS). We constructed a stably transfected mouse OS K7M2 cell line (shPTHR1‐ K7M2) using shRNA and knocked down AGT in these cells using siRNA‐AGT. The transfection efficiency and expression of AGT, chemokine C‐C motif receptor 3 (CCR3), and chemokine (C‐C motif) ligand 9 (CCL9) were determined using real‐time quantitative PCR. Cell viability and colony formation were assessed using Cell Counting Kit‐8 and crystal violet staining, respectively. Cell apoptosis and cycle phases were assessed by flow cytometry, and cell migration and invasion were evaluated using Transwell assays. Interference with PTHR1 upregulated the expression of AGT and CCR3, and downregulated that of CCL9, which was further downregulated by AGT knockdown. Cell viability, migration, invasion and colony formation were significantly decreased, while cell apoptosis was significantly increased in shPTHR1‐K7M2, compared with those in K7M2 cells (*P* < .05 for all). However, AGT knockdown further inhibited cell viability after 72 h of culture but promoted cell migration and invasion. PTHR1 interference decreased and increased the numbers of cells in the G0/G1 and G2/M phases, respectively, compared with those in K7M2 cells. Angiotensinogen knockdown increased the number of cells in the G0/G1 phase compared with that in the shPTHR1‐K7M2 cells. Therefore, PTHR1 affects cell viability, apoptosis, migration, invasion and colony formation, possibly by regulating AGT/CCL9 in OS cells.

## 1 INTRODUCTION

Osteosarcoma (OS) is the most prevalent malignant bone tumour affecting children, adolescents and young adults, and its onset manifests as pain and swelling in the affected bone, which disrupts sleep.^1^ The 5‐year survival rate for OS has significantly increased to 70% because of multi‐agent chemotherapy and surgical resection.^2^ However, ~20% of patients have clinically detectable metastases at presentation (usually in the lungs and bones), and survival rates are 19%‐30%.^3^ Therefore, the pathogenesis of OS should be explored in depth to develop novel therapeutic strategies for improving OS survival.

Parathyroid hormone type 1 receptor (PTHR1), a G‐protein‐coupled receptor, plays an important role in the occurrence and progression of OS.^4^ The expression of PTHR1 is upregulated in OS cells and tissues, particularly in metastatic tissues and samples from patients with recurrent OS samples.^4^, ^5^, ^6^ Studies have indicated that PTHR1, can be activated by its ligands, including parathyroid hormone (PTH) and parathyroid hormone‐related peptide (PTHrP), and can then exert tumour‐promoting effects.^7^, ^8^ Downregulating PTHR1 expression using shRNA inhibits OS cell proliferation and invasion, and reduces RANK ligand expression, thus suppressing tumour growth.^9^ Additionally, Li et al^10^ identified 1,163 differentially expressed genes (DEG) in tumour tissues from mice with PTHR1 knockdown and in tumour tissues from mice with control knockdown. A protein‐protein interaction (PPI) network revealed that angiotensinogen (AGT) was the hub node gene, and it interacted with CC chemokine receptor 3 (CCR3) and chemokine (C‐C motif) ligand 9 (CCL9) genes. However, interactions between PTHR1 and AGT remain unclear.

Angiotensinogen is the only precursor of angiotensin peptides and it is involved in the production of angiotensin II (AngII), which is the primary mediator of the renin‐angiotensin system (RAS).^11^ Angiotensin II participates in angiogenesis and regulates cell growth, apoptosis, migration, differentiation, extracellular matrix conformation and inflammation.^12^, ^13^, ^14^ Angiotensin II simulation activates the epithelial‐mesenchymal transition (EMT) in HepG2 cells.^15^ The overexpression of AGT induces inflammation via the JAK/STAT signalling pathway, eventually inhibiting A549 cell proliferation and promoting bronchopulmonary dysplasia.^11^ Thus, we speculated that downregulated AGT interacts with PTHR1 to reduce the inflammatory response and improve OS progression.

We investigated the effects of PTHR1 interference in mouse K7M2 OS cells and in a stably transfected shPTHR1‐K7M2 cell line based on the K7M2 OS line. We also assessed the effects of AGT knockdown on shPTHR1‐K7M2 cells. The findings should help to improve understanding of OS development and provide new targets for treating OS.

## 2 MATERIALS AND METHODS

### 2.1 Cell culture

The mouse OS K7M2 cell line (Cell Bank, Chinese Academy of Sciences, Shanghai, China) was cultured in Dulbecco's modified Eagle's medium (DMEM; Gibco) supplemented with 10% foetal calf serum (FBS, Gibco) and 1% penicillin/streptomycin (Gibco), and maintained in an incubator under a 5% CO_2_ atmosphere at 37°C. The cells were passaged after reaching 80%‐90% confluence.

### 2.2 Acquisition of lentivirus packaging and shPTHR1‐ K7M2 stable transfection cell lines

We constructed a PTHR1 interference vector using the empty lentiviral vector pLKO.1‐puro, and the following PTHR1 shRNA sequences: PTHR1 shRNA‐1, CCGGGCTACCAACTACTACTGGATTCTCGAGAATCCAGTAGTAGTTGGTAGCTTTTT; PTHR1 shRNA‐2, CCGGGAGTCTAAAGAGAACAAGGATCTCGAGATCCTTGTTCTCTTTAGACTCTTTTT; PTHR1 shRNA‐3, CCGGGCAATGTGACAAGCTGCTCAACTCGAGTTGAGCAGCTTGTCACATTGCTTTTT; PTHR1 shRNA‐4, CCGGCTCCAGCCATTGAGAACGAAACTCGAGTTTCGTTCTCAATGGCTGGAGTTTTT. Lentivirus packaging of PTHR1 shRNA vectors proceeded as described previously.^16^ Briefly, 293T cells (6 × 10^6^/well) were seeded into 6‐well plates, then cultured in complete medium for 24 h. The medium was replaced with complete medium without double resistance, then 100 μg recombinant PTHR1 shRNA vectors (PTHR1 shRNA‐1, shRNA‐2, shRNA‐3 and shRNA‐4) were transfected into the cells for 6 h using Lipofectamine 3000 (Thermo Fisher Scientific) as described by the manufacturer. The medium was replaced with complete medium, and viral supernatants collected 48 h later were centrifuged at 1000 rpm for 5 min. The supernatants were passed through 0.45‐μm PVDF filters, then centrifuged at 50 000 × g for 2 h at 4°C. The sediment was resuspended in 200 μL PBS and stored at −80°C.

Subsequently, K7M2 cells (6 × 10^5^/well) were seeded into 6‐well plates and cultured overnight. The medium was replaced with serum‐free medium, and the cells were infected with the packaged lentivirus supernatant (50 μL). After culture for 2 h, the medium was replaced with complete medium, which was removed after 48 h of infection, and replaced with medium containing puromycin (2 μg/mL) to select resistant cells.^17^ We then selected and expanded shPTHR1‐K7M2 cells in culture. Transfection efficiency was assessed by measuring *PTHR1* expression using real‐time quantitative PCR (RT‐qPCR). Table 1 shows the sequences of *PTHR1*.

**TABLE 1:** The sequences of all primers

Gene	Sequence (5’‐3’)
PTHR1	F: GTGGCAGTACCTTGTCCCG
	R: CGGTCAAATACCTCCCGTTC
AGT	F: TCTCCTTTACCACAACAAGAGCA
	R: CTTCTCATTCACAGGGGAGGT
CCR3	F: TCGAGCCCGAACTGTGACT
	R: CCTCTGGATAGCGAGGACTG
CCL9	F: CCCTCTCCTTCCTCATTCTTACA
	R: AGTCTTGAAAGCCCATGTGAAA
GAPDH	F: GGTGAAGGTCGGTGTGAACG
	R: CTCGCTCCTGGAAGATGGTG

### 2.3 Cell transfection

The siRNA‐angiotensinogen (si‐AGT) and siRNA‐negative control (si‐NC) cells (Yanzai Biotechnology (Shanghai) Co., Ltd.) were transfected as described option.^18^ The shPTHR1‐K7M2 cells were seeded in 6‐well plates and cultured overnight. The medium was then replaced with serum‐free medium, and the shPTHR1‐K7M2 cells were transfected with 20 nmol/L si‐AGT or si‐NC using Lipofectamine 300 (Thermo Fisher Scientific), essentially as described by the manufacturer. After 6 h of transfection, the serum‐free medium was replaced with complete medium and cultured for another 48 h. Transfection efficiency was determined by evaluating *AGT* expression by RT‐qPCR. Table 1 shows the sequences for *AGT*.

### 2.4 Cell viability and cloning assays

Cell viability was determined using Cell Counting Kit‐8 (CCK‐8; Beyotime Biotechnology) as described by the manufacturer. Harvested cells (1 × 10^4^) were seeded in 96‐well plates, cultured for 24, 48, 72 and 96 h, then the CCK‐8 reagent (10 μL) was added to the wells. Absorbance at 450 nm was measured 2 h later using a microplate reader.

We assessed colony formation by seeding cells into 6‐well plates and incubating them under a 5% CO_2_ atmosphere for 10 days. The supernatant was discarded when colonies became visible. The colonies were washed twice with PBS, then fixed in 4% paraformaldehyde at room temperature for 10 min. After two PBS washes, the colonies were stained with 0.5% crystal violet for 10 min, and visualized by microscopy.

### 2.5 Cell apoptosis and cycle assays

Cell apoptosis was determined using Annexin V‐FITC apoptosis assay kits (Beyotime Biotechnology) as described by the manufacturer. Briefly, the cells were resuspended in Annexin V‐FITC binding buffer (195 μL), then 5 μL of Annexin V‐FITC was added, followed by 10 μL of propidium iodide (PI), and the mixture was incubated in the dark for 20 min. The cells were analysed by flow cytometry, and apoptosis rates were calculated.

Cell cycle phases were also determined by flow cytometry. Cells were resuspended in PBS (200 μL), then chilled 70% ethyl alcohol (4 mL) was added. The cells were fixed at 4°C overnight, centrifuged at 1000 × g for 5 min, then washed with PBS and centrifuged. The sediment was incubated at 37°C for 30 min in PBS containing RNase (50 μg/mL), followed by PI (final concentration, 50 μg/mL) in the dark for 30 min. Cell cycles phases were determined using flow cytometry within 24 h.

### 2.6 Cell migration and invasion assays

Cell migration and invasion of cells were evaluated using Transwell chambers (pore size 8 μm; Guangzhou Jet Bio‐Filtration Co., Ltd.). Transwell chambers were coated with matrix glue to assay cell invasion. Cells were inoculated into the upper inserts of Transwell chambers, and medium with 10% FBS was added into the lower chambers. The cells were incubated for 48 h, fixed in 4% paraformaldehyde for 20 min, washed with PBS, and then incubated with crystal violet for 20 min. Excess stain was removed, and relative cell numbers were analysed on microscopy images.

### 2.7 RT‐QPCR

Total RNA was extracted from K7M2, shPTHR1‐K7M2 and siAGT‐shPTHR1‐ K7M2 cells using RNAiso Plus (TRIzol; Takara Bio Inc, Kusatsu, Japan) as described by the manufacturer. The purity and concentration of total RNA were determined using a microplate reader. Isolated RNA was reverse transcribed into cDNA using PrimeScript™ RT Master Mix (Takara Bio Inc) at 37°C for 60 min and 85°C for 5 s. The cDNA was amplified by qPCR using 2x SYBR Premix EX Taq (Thermo Fisher Scientific Inc) at 95°C for 3 min, followed by 40 cycles of 95°C for 10 s, and 60°C for 30 s. Table 1 shows the sequences of the included primers, and *GAPDH* was the housekeeping gene. The relative mRNA expression of *PTHR1*, *AGT*, *CCR3* and *CCL9* was calculated using the 2^−ΔΔCt^ method.^19^

### 2.8 Statistical analysis

All experiments were conducted in triplicate, and data are expressed as mean ± standard deviation (SD). All data were statistically analysed using GraphPad prism 5 (GraphPad Software Inc). Multiple groups were compared using one‐way analyses of variance (ANOVA) followed by Tukey tests. Student t‐tests was used for comparisons between two groups. Values with *P* < .05 were considered statistically significant.

## 3 RESULTS

### 3.1 Screening stably transfected shPTHR1‐K7M2 cells and effects of PTHR1 interference on *AGT*, *CCR3* and *CCL9* expression

**Figure 1:**
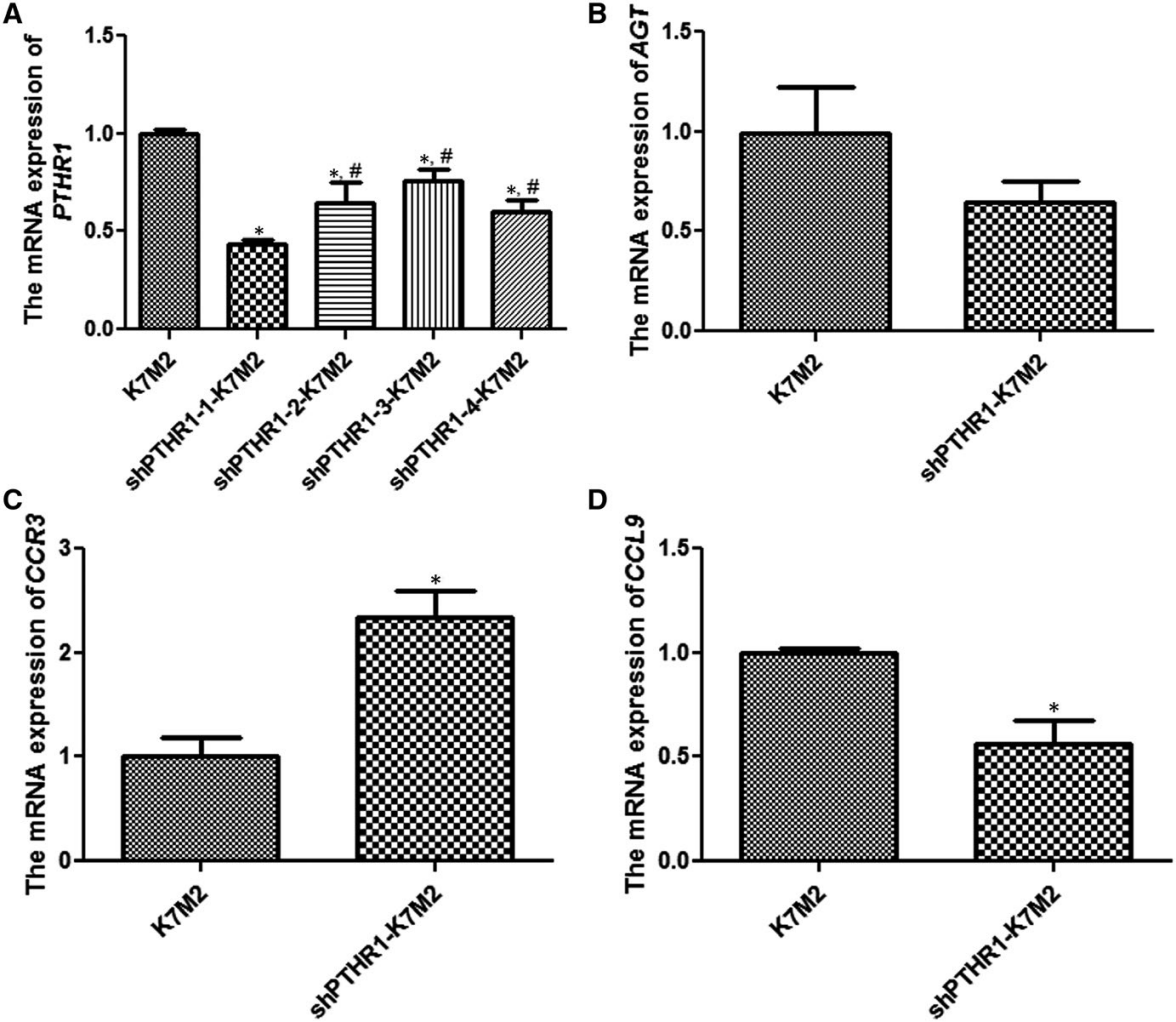
Screen of shPTHR1‐ K7M2 stably transfected cell lines and effects of PTHR1 interference on *AGT*, *CCR3* and *CCL9* expression. (A) The Expression of *PTHR1* mRNA determined by real‐time quantification PCR (RT‐qPCR). *: *P* < .05 vs. K7M2 cells; ^#^: *P* < .05 vs. shPTHR1‐1‐K7M2 cells. Expression of *AGT* (B), *CCR3* (C) and *CCL9* (D) mRNA determined by RT‐qPCR. *: *P* < .05 vs. K7M2 cells

Stably transfected cell lines were screened using puromycin, and *PTHR1* expression in the cells was determined by RT‐qPCR. Compared with the K7M2 group, the expression of *PTHR1* was significantly decreased after transfection with PTHR1 shRNA‐1, shRNA‐2, shRNA‐3 and shRNA‐4 (*P* < .05, Figure 1A). The expression of *PTHR1* was significantly lower after transfection with PTHR1 shRNA‐1 compared with the other groups (*P* < .05, Figure 1A). Therefore, we selected K7M2 cells that were stably transfected with PTHR1 shRNA‐1 for subsequent experiments.

Levels of *AGT*, *CCR3* and *CCL9* expressed in K7M2 and shPTHR1‐K7M2 cells were compared using RT‐qPCR. The expression of *AGT* was significantly upregulated in shPTHR1‐K7M2, compared with K7M2 cells (*P* < .05, Figure 1B). The expression of *CCR3* was significantly upregulated after PTHR1 interference compared with K7M2 cells (*P* < .05, Figure 1C). The trend of *CCL9* expression was inverse to that of *CCR3* expression (Figure 1D).

### 3.2 Effects of PTHR1 interference on K7M2 cell viability, apoptosis, and cycle phase

**Figure 2:**
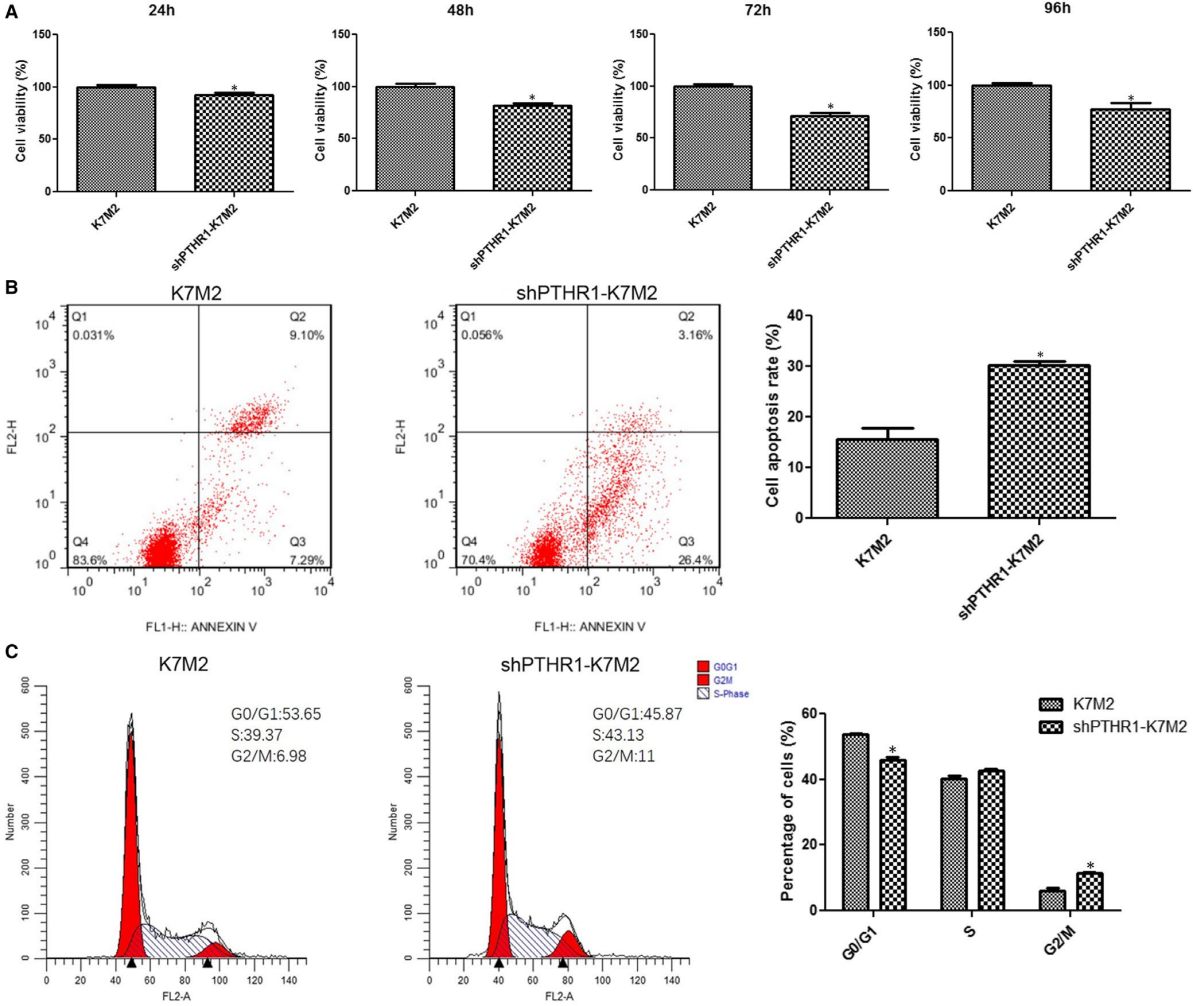
Effects of PTHR1 interference on cell viability, apoptosis and cycle phase. (A) K7M2 and shPTHR‐K7M2 cell viability after incubation for 24, 48, 72 and 96 h. Apoptosis (B) and cycle phases (C) of K7M2 and shPTHR‐K7M2 cells determined by flow cytometry. *: *P* < .05 vs. K7M2 cells

The viability of shPTHR1‐K7M2 cells at 24, 48, 72 and 96 h was significantly inhibited (*P* < .05, Figure 2A) and the apoptosis rate was significantly increased (*P* < .05, Figure 2B) compared with K7M2 cells. The distribution of K7M2 and shPTHR1‐K7M2 cells in S phase did not significantly differ (*P* > .05, Figure 2C). After PTHR1 interference, the numbers of cells in the G0/G1 and G2/M phases were significantly decreased and increased, respectively, compared with K7M2 cells (*P* < .05, Figure 2C). These results showed that PTHR1 interference inhibited K7M2 cell viability and promoted apoptosis by affecting the cell cycle.

### 3.3 Effects of PTHR1 interference on the cell migration, invasion and colony formation in K7M2 Cells

**Figure 3:**
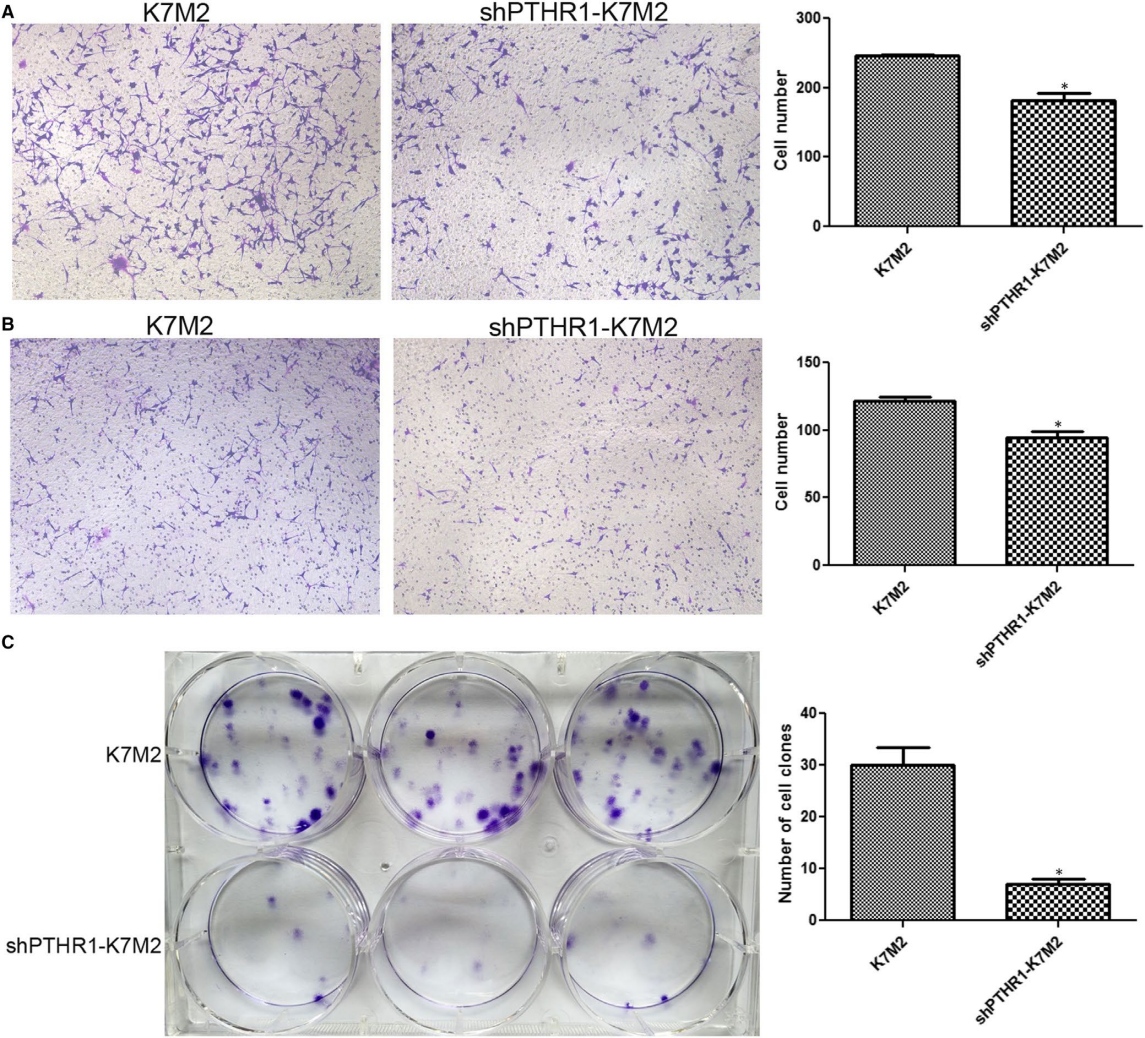
Effects of PTHR1 interference on cell migration, invasion and colony formation. K7M2 and shPTHR‐K7M2 cell migration (A) and invasion (B) assessed using Transwells. (C) Colony formation by K7M2 and shPTHR‐K7M2 cells. *: *P* < .05 vs. K7M2 cells

Cell migration and invasion assessed using Transwells showed that the numbers of cells were significantly decreased after transfection with shPTHR1 compared with K7M2 cells (*P* < .05, Figure 3A and B). These results suggested that the migration and invasion of shPTHR1‐K7M2 cells were inhibited compared with K7M2 cells. Furthermore, significantly fewer colonies were generated by shPTHR1‐K7M2, than K7M2 cells (*P* < .05, Figure 3C). This indicated that PTHR1 interference restrained the formation of cell colonies.

### 3.4 *AGT* knockdown in the shPTHR1‐K7M2 cells and the expression of related genes

**Figure 4:**
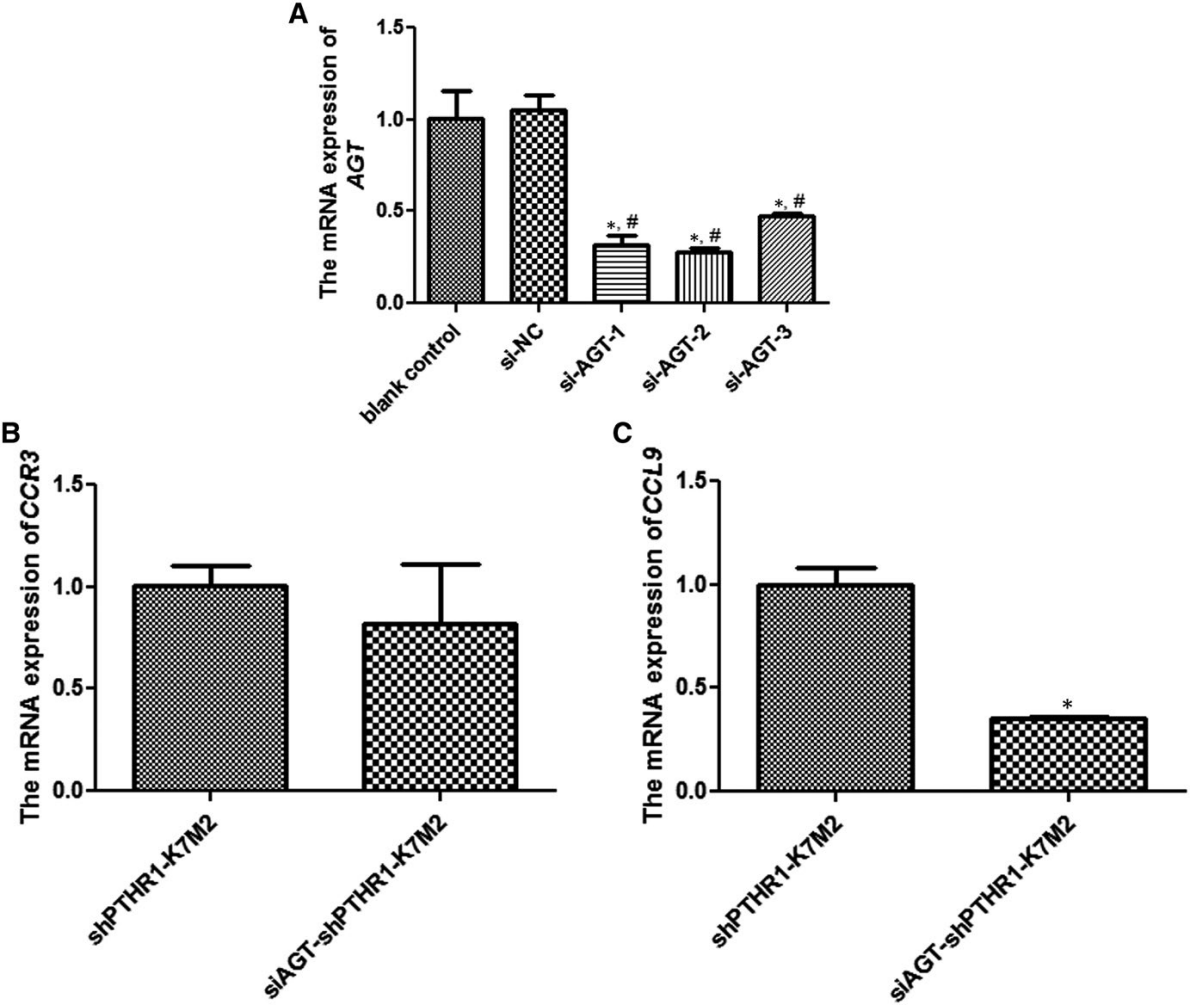
Knockdown of *AGT* in shPTHR1‐K7M2 cells and expression of *CCR3* and *CCL9*. (A) Expression of *AGT* mRNA determined by RT‐qPCR. *: *P* < .05 vs. blank control^#^: *P* < .05 vs. si‐NC. Expression of *CCR3* (B) and *CCL9* (C) mRNA*: *P* < .05, vs. shPTHR1‐K7M2 cells

We further explored the relationship between *AGT* and *PTHR1* by knocking down the *AGT* gene in shPTHR1‐K7M2 cells. The expression of *AGT* did not significantly differ between the blank control and si‐NC groups (*P* > .05, Figure 4A). Transfection with si‐AGT‐1/2/3 significantly decreased *AGT* mRNA expression compared with the si‐NC group (*P* < .05, Figure 4A). These results confirmed that *AGT* was appropriately knocked down in shPTHR1‐K7M2 cells.

Levels of *CCR3* expression did not significantly differ between shPTHR1‐K7M2 cells with and without *AGT* knockdown (*P* > .05, Figure 4B), whereas *CCL9* expression was significantly downregulated in shPTHR1‐K7M2 cells with *AGT* knockdown (*P* < .05, Figure 4C).

### 3.5 Effects of *AGT* knockdown on shPTHR1‐K7M2 cell viability, apoptosis and cycle phase

**Figure 5:**
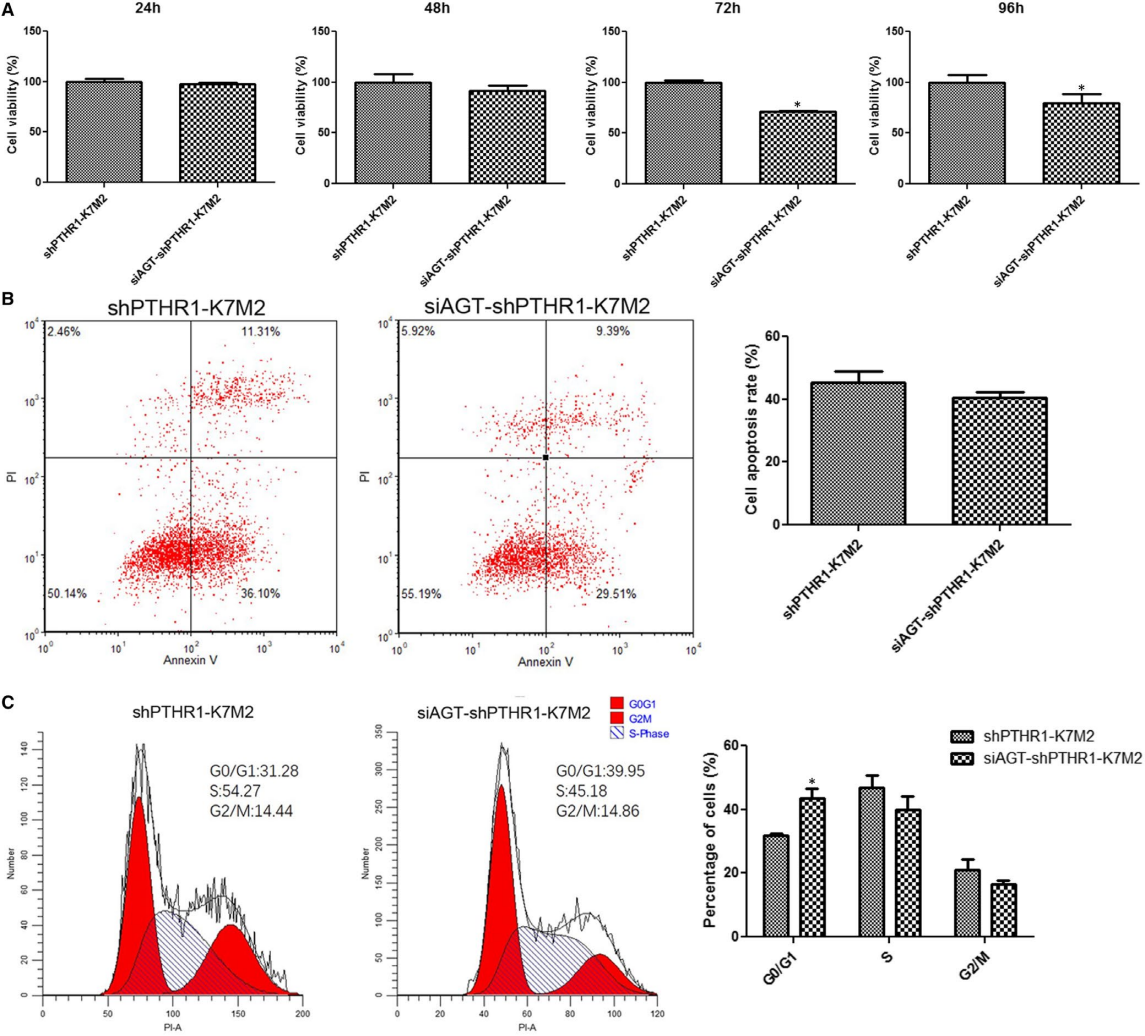
Effects of *AGT* knockdown on cell viability, apoptosis and cycle phases. (A) Viability of shPTHR‐K7M2 cells with and without *AGT* knockdown after incubation for 24, 48, 72 and 96 h. Apoptosis (B) and cycle phases (C) of shPTHR‐K7M2 cells with and without *AGT* knockdown. *: *P* < .05, vs. shPTHR1‐K7M2 cells

The viability of shPTHR1‐K7M2 cells with and without *AGT* knockdown did not significantly differ after incubation for 24 and 48 h (*P* > .05, Figure 5A), but became significantly suppressed in shPTHR1‐K7M2 cells with knockdown after incubation for 72 h and 96 h (*P* < .05, Figure 5A). Apoptosis rates did not significantly differ between shPTHR1‐K7M2 cells with and without *AGT* knockdown (*P* > .05, Figure 5B). The number of cells in the G0/G1 phase was significantly increased in shPTHR1‐K7M2 cells with *AGT* knockdown. (*P* < .05, Figure 5C), but the distribution of the S and G2/M phases did not significantly differ between shPTHR1‐K7M2 cells with and without *AGT* knockdown (*P* > .05, Figure 5C).

### 3.6 Effects of *AGT* knockdown on the shPTHR1‐K7M2 cell migration, invasion and colony formation

**Figure 6:**
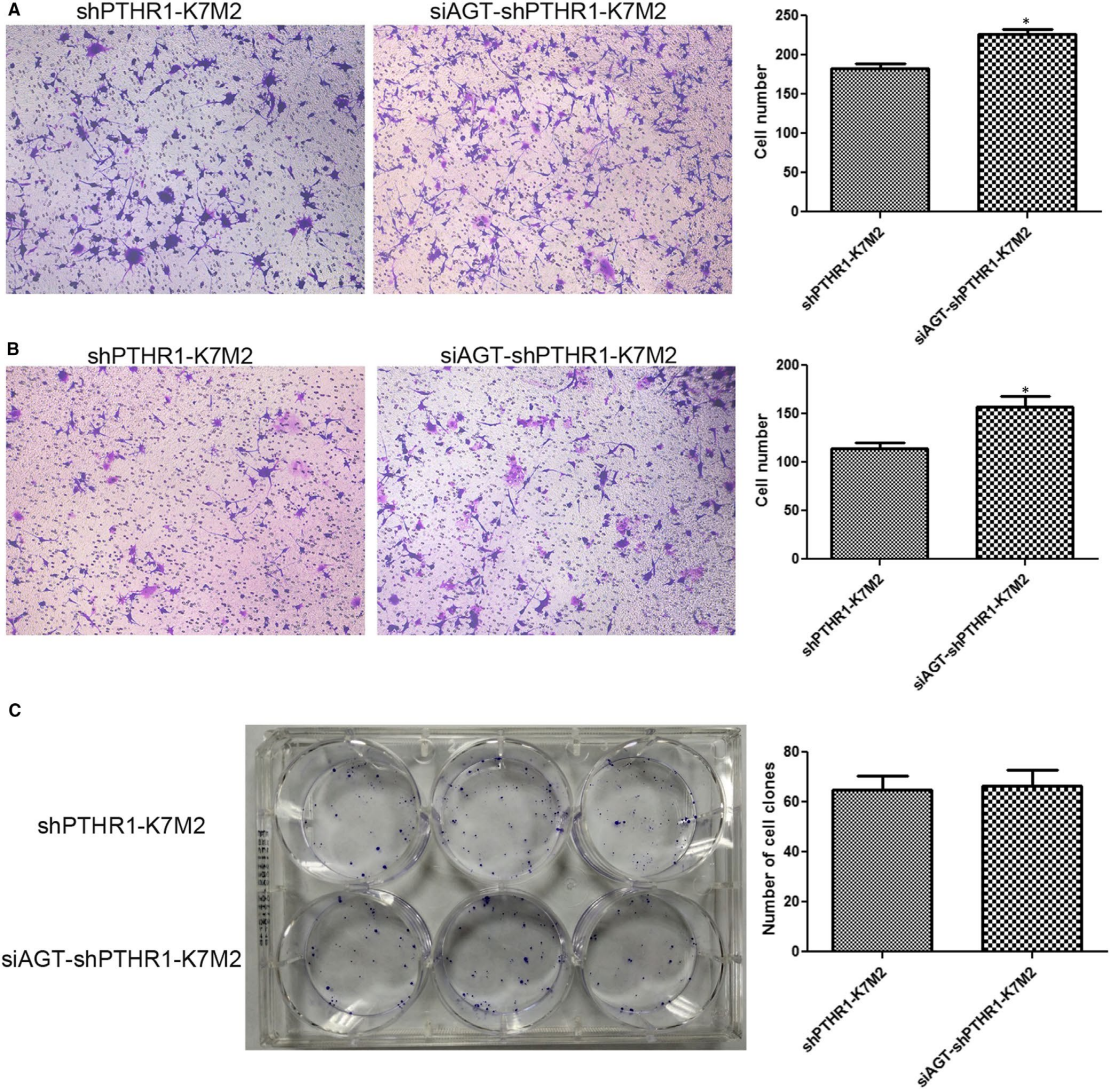
Effects of *AGT* knockdown on cell migration, invasion and colony formation. Migration (A), invasion (B) and colony formation (C) of shPTHR‐K7M2 cells without and with *AGT* knockdown. *P < .05 vs. shPTHR‐K7M2

The Transwell results showed that compared with the shPTHR1‐K7M2 cells, the number of shPTHR1‐K7M2 cells with *AGT* knockdown was significantly increased (*P* < .05, Figure 6A,B). These results indicated that *AGT* knockdown enhanced shPTHR1‐K7M2 cell migration and invasion. In addition, the numbers of colonies formed did not significantly differ between the shPTHR1‐K7M2 cells with and without *AGT* knockdown (*P* > .05, Figure 6C).

## 4 DISCUSSION

OS is the most prevalent primary malignancy of the skeletal system. It is characterized by aggressive clinical development and high metastatic potential, and is the leading cause of death in adolescents.^20^ Previous studies have indicated that PTHR1 interacts with AGT, and that PTHR1 expression is associated with the progression of OS.^6^, ^10^, ^21^ We created K7M2 stable transfection cell lines with PTHR1 knockdown using shRNA, then knocked down AGT in these lines using siRNA‐AGT. Viability, migration, invasion and colony formation were all significantly decreased while cell apoptosis was evidently increased in the stably transfected shPTHR1‐K7M2 cells, compared with normal K7M2 cells. However, the viability of shPTHR1‐K7M2 cells with *AGT* knockdown was further inhibited, whereas their migration and invasion were promoted after culture for 72 h compared with the shPTHR1‐K7M2 cells without *AGT* knockdown, whereas apoptosis and colony formation did not significantly differ between the two cell lines. The PTHR1 interference decreased the number of cells in the G0/G1 phase and increased the number of cells in G2/M phase compared with normal K7M2 cells, while AGT knockdown increased the number of cells in the G0/G1 phase compared to that in shPTHR1‐K7M2 cells without AGT knockdown. In addition, PTHR1 interference upregulated the expression of *AGT* and *CCR3*, and downregulated that of *CCL9*; and AGT knockdown further downregulated *CCL9* expression.

Parathyroid hormone (PTH) regulates calcium homeostasis and bone development, and paracrine/autocrine PTH‐associated protein (PTHrP) plays a central role in endochondral bone formation and remodelling.^22^ PTHR1, the common PTH/PTHrP receptor, PTHR1 plays an important role in the development of OS by regulating angiogenesis, inflammatory pathway genes, and Wnt.^23^ Our results showed that PTHR1 interference suppressed K7M2 cell viability, migration, invasion and colony formation, while accelerating cell apoptosis. In addition, PTHR1 interference decreased the numbers of cells in the G0/G1 phase and increased the number of cells in the G2/M phase. Li et al, have shown that that β‐alanine‐mediated PTHR1 downregulation inhibits cell proliferation, migration, U2OS cell invasion and tumour formation.^24^ Mangiferin downregulates PTHR1 expression in OS cells, effectively suppresses the growth, and induces the apoptosis of OS cells.^25^ These combined with the present results indicate that PTHR1 interference influences OS progression by inhibiting cell viability, migration, invasion and colony formation, and by inducing cell apoptosis through mediating cells in the G0/G1 and G2/M phase.

To further investigate interactions between PTHR1 and AGT, we knocked down AGT in shPTHR1‐K7M2 cells and examined their growth. The expression of AGT was significantly upregulated by PTHR1 interference in K7M2 cells, whereas transfection with si‐AGT decreased AGT expression. Knocking down AGT further inhibited the viability, but increased the migration and invasion of shPTHR1‐K7M2 cells after incubation for 72 h, and increased the number of cells in the G0/G1 phase. Angiotensinogen belongs to the serpin family, and it inhibits the proliferation and migration of endothelial cells in vitro, capillary tube formation, and neovascularization.^26^ The overexpression of AGT could lead to the apoptosis of alveolar epithelial cells and pulmonary fibrosis.^27^ Furthermore, adenovirus‐mediated AGT overexpression inhibits tumour growth by 70% in established human MDA‐MB‐231 breast cancer cells compared with controls, and blocks the tumorigenesis and lung metastasis of MDA‐MB‐231 and mouse melanoma B16F10 cells in C57BL/6 mice.^28^ Therefore, we speculated that PTHR1 regulates cell growth and induces cell apoptosis by interacting with AGT.

The interactive genes of AGT (*CCR3* and *CCL9*) were further determined by RT‐qPCR. The hybrid G‐protein‐coupled receptor CCR3 interacts with various inflammatory chemokines, including high‐affinity agonist eotaxin‐1 (CCL11), eotaxin‐2 (CCL24), eotaxin‐3 (CCL26) and CCL5.^29^ The processes of many pathological states such as Crohn's disease,^30^ neurodegenerative disease,^31^ glioblastoma ^32^ and eosinophilic myocarditis ^33^ involve CCR3 signalling. Matsuo et al ^34^ showed that Ephedra Herb could improve Th2‐mediated anaphylactic disease by inhibiting chemotaxis mediated by CCR3, CCR4 and CCR8. The CCL9 protein is involved in inflammatory responses and immunological processes process.^35^ High levels of CCL9 are induced in Gr‐1 + CD11B+ immature myeloid cells and in tumour‐bearing mice before lung metastasis, and knockdown of CCL9 in bone marrow cells reduces the survival and metastasis of tumour cells.^36^ We showed here that *CCR3* expression was upregulated, and *CCL9* expression was downregulated in cells with PTHR1 interference, and that AGT knockdown did not significantly affect *CCR3* expression, but further downregulated *CCL9* expression. These results indicated that PTHR1/AGT knockdown affects OS cell growth by regulating *CCL9* expression.

This study had several limitations. The relationship between PTHR1 and CCL9 requires verification by a rescue experiment, and AGT, CCR3 and CCL9 protein expression should be assessed by western blotting. Our findings also require confirmation in animal models in vivo. Further studies of preclinical or clinical models are required. In conclusion, PTHR1 interference might inhibit cell viability, migration, invasion and colony formation and promote cell apoptosis by regulating AGT/CCL9 in OS cells, thus improving OS. Our findings will help to enhance knowledge of OS pathogenesis and provide a theoretical basis for the treatment of OS using PTHR1 and AGT as therapeutic targets.

## CONFLICT OF INTEREST

The authors declare that the research was conducted in the absence of any commercial or financial relationships that could be construed as a potential conflict of interest.

## AUTHOR CONTRIBUTIONS

Shenglong Li and Yaohua Shang designed the experiments; Fei Liu, Yi Pei and Yujin Dong performed the experiments; Shenglong Li wrote the paper; all authors discussed the results and contributed to the modification of the manuscript.

## Data Availability

The data in the current study are available from the corresponding authors upon reasonable request.
